# Transforming research to improve therapies for trauma in the twenty-first century

**DOI:** 10.1186/s13054-024-04805-6

**Published:** 2024-02-13

**Authors:** Nicole P. Juffermans, Tarik Gözden, Karim Brohi, Ross Davenport, Jason P. Acker, Michael C. Reade, Marc Maegele, Matthew D. Neal, Philip C. Spinella

**Affiliations:** 1 Department of Intensive Care, Erasmus Medical Center, Rotterdam, The Netherlands; 2 Laboratory of Translational Intensive Care, Erasmus Medical Center, Rotterdam, The Netherlands; 3 Centre for Trauma Sciences, Blizard Institute, Queen Mary University of London, London, UK; 4 Canadian Blood Services, Innovation and Portfolio Management, Edmonton, AB Canada; 5 Department of Laboratory Medicine and Pathology, University of Alberta, Edmonton, AB Canada; 6 Medical School, University of Queensland, Brisbane, QLD Australia; 7 Department of Intensive Care Medicine, Royal Brisbane and Women’s Hospital, Brisbane, QLD Australia; 8 Department of Trauma and Orthopedic Surgery Cologne-Merheim Medical Center Institute of Research, Operative Medicine University Witten-Herdecke, Cologne, Germany; 9 Trauma and Transfusion Medicine Research Center, Department of Surgery, University of Pittsburgh, Pittsburgh, PA USA

**Keywords:** Trauma, Research methodology, Therapies, Future of care

## Abstract

Improvements have been made in optimizing initial care of trauma patients, both in prehospital systems as well as in the emergency department, and these have also favorably affected longer term outcomes. However, as specific treatments for bleeding are largely lacking, many patients continue to die from hemorrhage. Also, major knowledge gaps remain on the impact of tissue injury on the host immune and coagulation response, which hampers the development of interventions to treat or prevent organ failure, thrombosis, infections or other complications of trauma. Thereby, trauma remains a challenge for intensivists. This review describes the most pressing research questions in trauma, as well as new approaches to trauma research, with the aim to bring improved therapies to the bedside within the twenty-first century.

## Introduction

Worldwide, trauma results in death in over 6 million people and causes lasting disability in many more [1]. Trauma is the leading cause of death in the young, but its impact in the elderly is also rising. Patterns of death appear to differ across the globe [2, 3]. These differences are attributed partly to differences in receipt of immediate treatment of shock and coagulopathy, and partly to differences in the proportion of people of older age and/or with comorbidities. These differences in trauma trajectory also highlight the impact of both acute and longer term complications on the outcome of trauma.

Despite major advances in resuscitation of trauma over the last 20 years, our knowledge of the pathophysiology of traumatic bleeding remains incomplete. Besides bleeding, tissue injury triggers a complex dysregulated host immune and coagulation response, which contributes to longer term complications and late mortality. If traumatic brain injury (TBI) is involved, mortality is even worse. TBI has also long-term consequences in survivors, including chronic impairment and late-onset neurodegeneration, all of which contribute to high socio-economic burden [4]. Every year, there are more than 27 million new TBI cases with the expectation that numbers are likely to rise in the future [5, 6].

**Fig. 1:**
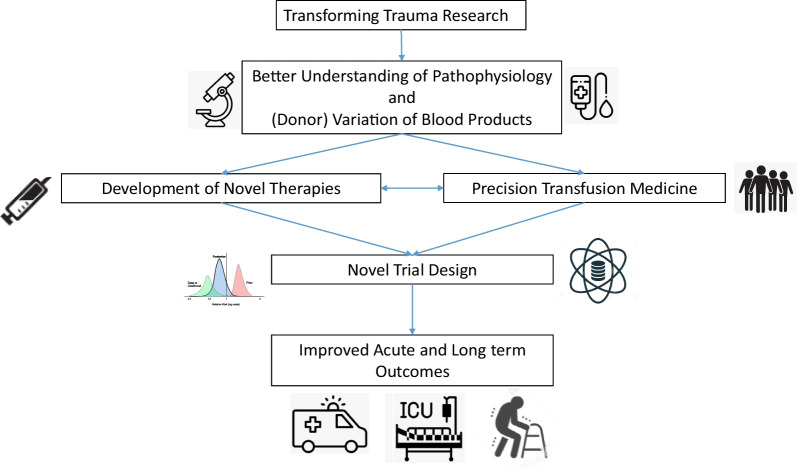
A translational approach to improve therapies for trauma

These numbers call for a need to improve care for trauma patients, who arrive at the hospital at all times, with little or no time to prepare. Institutional trauma teams with clear leadership, role assignment and organization have been shown imperative for optimizing delivery of acute care, aided by resuscitation protocols and use of transfusion packs. But in order to further improve, additional steps are needed. This review aims to describe what is needed to advance research with the aim to bring improved or novel therapies to the bedside of trauma patients in the near future (Fig. 1). The outcome of trauma is determined by treatment of the initial shock phase but also by TBI and by complications occurring in a later phase, including a dysregulated host response contributing to nosocomial infections and (micro)thrombo-embolic events. Thereby, the scope of this review describes what is needed to improve initial resuscitation as well as longer term care.

## Initial care: understanding the pathophysiology of traumatic bleeding

Our current understanding is that an acute coagulopathy of trauma develops early after injury that is driven by a combination of tissue damage and systemic hypo perfusion. We also know that coagulopathy develop due to our approaches to resuscitation. These coagulation deficits are associated with early deaths from hemorrhage, and later deaths and complications, such as multiple organ dysfunction and thrombotic events [7].

However, many gaps remain. We do not understand the very early stages of initiation of acute traumatic coagulopathy, how it evolves in severity and complexity in the face of further bleeding and massive transfusions, or when and how it transitions to a prothrombotic state in early survivors. By the time patients arrive in hospital, coagulopathy is already a complex mix of hyperfibrinolysis, hypofibrinogenaemia, anticoagulation, platelet and endothelial dysfunction. The balance and severity of these depends on multiple factors including severity of injury, type of injuries, speed and depth of shock, endogenous catecholamine release and others [8]. Detailed elucidation of each aspect of coagulopathy at all these time points is critical if we are to develop effective treatments for major hemorrhage.

Currently, apart from the antifibrinolytic tranexamic acid, all coagulopathy treatments are based on the presumption that coagulation factors are consumed or inhibited, and that replacement of their substrates is both appropriate and effective [9]. Replacement with plasma may be the prime example of this, but the mechanism of action of plasma is not well understood. Nor is it known whether substituting specific components present in the plasma are a better option than the whole product, as transfusion of plasma also means supplementing anticoagulant factors. In addition, it is not known whether specific plasma products are superior to others. With regard to platelets, replacement with donor platelet transfusion may be beneficial by overcoming dysfunctional platelets, but the optimal timing, storage conditions or dose is not known.

Of note, it is possible that benefit of replacing a loss (of function) of factors and platelets is limited, as we know that many of the processes occurring after severe trauma are likely to inactivate these treatments, as they do with the endogenous factors [10]. Conversely, there are no specific treatments that inhibit the driving processes of traumatic coagulopathy. The dysregulated activation of protein C is central to traumatic coagulopathy [11]. Synthetic and natural inhibitors of the activated protein C pathway exist and are targets for future research. The impact of other anticoagulant proteins is also not well explored. At present, there is no coordinated programme to explore drug discovery in these areas for trauma patients.

Current treatments that are used for replacement therapy are generally administered empirically as part of major hemorrhage protocols. This practice assumes that all patients who receive them will either benefit or at minimum will not be harmed in the process. There is increasing evidence that an empirical approach of substituting concentrated products such as fibrinogen or prothrombin complex concentrates [12, 13] in an undifferentiated way may not confer benefit. In major hemorrhage, there is a movement toward precision delivery of coagulation therapies, however the success of this approach depends not only on rapid, accurate diagnostics, but also on the ability to rapidly administer effective treatment. This requires both better understanding of the pathophysiology, and the development of novel diagnostics which can rapidly detect specific derangements that can corrected by targeted therapeutics. Recent evidence suggests that heterogeneity of treatment effect occurs with transfused blood components, and only patients with very specific endotypes may benefit from our current resuscitation practices [14, 15].

Many of the immediate and downstream consequences of traumatic hemorrhage are due to prolonged and severe cell and organ ischemia. This leads to endothelial injury, myocardial death, multiple organ dysfunction and cerebral injury among others which require specific research focus in the context of major trauma. Time and distance will always be barriers to good outcomes for trauma patients unless we can develop new ways to mitigate the effects of ischemia.

Finally, the coagulation system is also part of the body’s mechanisms that sense and respond to injury. There are hundreds and possibly thousands of other damage molecules released passively and actively following injury which are sensed by the immune system and trigger the injury response [16]. These lead to complex system-wide activation of the transcriptome which has multiple downstream consequences [15]. Dysregulated or inappropriate responses can dramatically affect clinical outcomes such as organ dysfunction or immunosuppression [17]. We are only at the beginning of unpicking these complex changes which will be important for whole-system mechanistic understanding and new diagnostic and therapeutic approaches. Table 1 summarizes some key priorities in understanding traumatic hemorrhage.

**Table 1:** Priorities in research to improve trauma therapies in poly trauma

*Acute care*	
Understand (trajectory of) coagulopathy, including the endogenous processes that inactivate coagulation factors and platelet function	
Investigate dysregulated immune response	
Investigate ways to delay ischemia	
Identify point of care biomarkers and diagnostics of dysregulated pathways	
Development of a drug discovery programme which (at least) includes blocking of activated protein C and scavenging of damage molecules	
Understand the impact of blood product manufacturing methods, storage conditions and donor characteristics on outcome of trauma	
Development of a programme aimed at standardization of product manufacturing and storage	
*Longer term care*	
Investigate biomarker-guided use of prophylactic antibiotics	
Investigate optimal dose and timing of thrombo-prophylaxis	
Identify cost-effective rehabilitation programmes	
*Research methodology*	
Use of translational models that reflect clinical trauma	
Implement acceptation of exception from informed consent	
Establish and implement use of a core outcome set	
Improve quality of trauma registries including physiologic data	
Use of adaptive trial designs to include platform trials	
Use of Bayesian analyses	

## Initial care: understanding the impact of blood product quality

In addition to understanding the unique response of each patient to blood products, variation in the products themselves warrants close attention. Each blood product is influenced by a number of factors, including donor characteristics, component manufacturing methods, post-production processing, and storage factors. This means that no two blood products in the blood bank are the same. Differences in production processes exist across different countries, and even within a single organization [18]. While regulatory standards are applied, in part to address these concerns, current standards are inconsistent or ill-defined in terms of component manufacturing. For example, [19–21] current Canadian standards for blood manufacturing hold that the hemoglobin content of a red blood cell concentrate (RBC) may range between 30 and 90 g; a fact that transfusing clinicians are generally unaware of. Other factors, such as the method used to separate blood components from whole blood [20, 22–24] blood bag materials, pre-processing hold times and temperatures, centrifugation conditions, the additive solutions used [25, 26] and pre-storage leukoreduction [27] have all been shown to affect the quality characteristics of transfused products. In addition, donor factors such as the donor sex, age, ethnicity, frequency of donation, and pre-donation hemoglobin also affect the quality characteristics of the blood products [21, 28–31].

Given all these factors, it has been very difficult to achieve any level of global, or even national, standardization of blood products. As a consequence, comparisons of products in Canada, the USA and Europe have highlighted that the products distributed for transfusion are not equivalent [20, 23, 32, 33]. Collectively, these studies show that there are significant differences in the levels of hemolysis, potassium, cytokine and microparticle levels, oxidative stress, oxygen carrying capacity, deformability, and residual plasma, platelet and leukocyte concentrations across the different methods used to manufacture an RBC product. Similar studies have been performed for platelet [34, 35] and plasma [36] components highlighting the variability that exists in “what is in the bag”.

And ‘what is in the bag’, is a relevant question. In observational studies, the method of whole blood processing was associated with in hospital mortality of transfused adults [37]. Patients who received fresh RBCs (≤ 7 days of storage) prepared by a whole blood filtration, top / top manufacturing method had a higher risk of in-hospital mortality than those transfused with mid-age RBCs (stored 8–35 days) prepared by the red cell filtration (top/bottom) method. Similarly, RBC component-specific factors such as additive solution and collection method have been associated with differences in post-transfusion hemoglobin increments [38] and venous thromboembolism [39]. These works suggest that adverse transfusion outcomes might be reduced by making changes to blood processing methods and inventory management practices. Understanding these relations in trauma patients is there for a research priority (Table 1).

## Longer term care: how to improve anti-inflammatory and anti-thrombotic treatment

After surviving the initial insult, a proportion of trauma patients die later in the course of trauma [40]. Organ failure, thrombosis and infections are likely to contribute to adverse outcome. Most studies report that sepsis (often with multiple organ failure) is the commonest cause of late death after trauma [41]. Dysregulated inflammation (either pro- or anti-inflammatory [42]) places trauma patients at risk of nosocomial infection, and makes infection difficult to distinguish from the physiological response to trauma. Hence, a pressing question is when to start antibiotics. Several biomarkers that might assist this decision have been evaluated: e.g. procalcitonin, C-reactive protein, glycosylated hemoglobin, neutrophil/lymphocyte ratio, interleukin-17, caspase-1, vanin-1, high-density lipoprotein, and thrombin-activable fibrinolysis inhibitor. No trial has yet demonstrated that biomarker-guided treatment improves patient outcomes (although a small trial of procalcitonin showed promise [43]). Prophylactic antibiotics are indicated after many forms of major trauma, but these risk bacterial resistance. Despite convincing evidence that prolonged antibiotic prophylaxis offers no benefit [44], practice surveys show many clinicians continue antibiotics > 72 h [45]. Research seeking more effective ways to harmonize evidence with practice is required. Selective decontamination of the digestive tract using non-absorbable antibiotics (SDD) has a significant benefit on infection outcomes in small randomized trials in trauma patients in parts of the world with low antibiotic resistance. Also, in a nearly 6000 patient trial in general ICU patients, the proportion of patients with a trauma diagnosis had a numerical trend toward benefit with the use of SDD [46].

A hypocoagulable state that frequently occurs in early trauma can progress to a hypercoagulable state within minutes to days, driven by injury-induced platelet activation, excess thrombin, and a surge in plasminogen activator inhibitor-1 activity that precipitates fibrinolytic shutdown [8]. Venous thrombosis is reported in 2–5% of trauma patients overall, but up to 44% in high-risk patients despite thrombo-prophylaxis with low molecular weight heparin (LMWH) [47]. Pulmonary embolism is the third leading cause of death among patients who survive the first 24 h after trauma [47]. The main knowledge gaps are when to initiate LMWH, and at what dose. While large observational series note earlier (< 24 h) initiation is associated with lower mortality [48], this question will almost certainly best be answered by tailoring treatment to individual patients. Commencing LMWH once standard laboratory tests have normalized misses the greatest risk period of thrombo-embolic events. Currently no diagnostic test paired with an intervention has been shown to improve outcomes and hence research should focus on a potential role for viscoelastic tests, or other coagulation tests.

Up to 30% of survivors of the acute phase of severe trauma have persistent disability and reduced quality of life [39]. It is recognized that the persistent inflammation immunosuppressive catabolism syndrome (PICS) is associated with ongoing protein catabolism with loss of functional recovery, as well as with immune suppression, including a reduced generation of cytokines, loss of monocyte-macrophage function, and a reduction in the number and function of effector T cells. These alterations are associated with poor wound healing, infections, a high late mortality and a protracted recovery in those who survive [49]. Optimizing functional recovery in survivors of severe trauma has received comparatively little attention. Following musculoskeletal trauma, the bed rest and immobilization of previous decades is now recognized to lead to unwarranted weakness, stiffness, chronic pain and functional decline [50]. Consequently, multidisciplinary rehabilitation programs involving psychology, nutrition, physiotherapy, and pain medicine are considered best practice. There is evidence supporting this strategy in elderly patients with mild trauma [51], but with the exception of severe TBI [52], few trials have been performed in complex trauma [53]; therefore an evidence gap needing to be filled. As innate and adaptive immune responses can differ depending on age, comorbidities and injury pattern, knowledge may be derived with the use of bioinformatics tools using data from large registries, which may improve the phenotyping of injury patterns. Of note, a basic challenge in many nations appears to be providing access to this care. For example, only 13–25% of patients who survive moderate or severe trauma receive comprehensive interdisciplinary inpatient rehabilitation [54], with most discharged from acute hospitals to residential care facilities that have little or no rehabilitation available. How can cost-effective rehabilitation be demonstrated? Key questions to advance longer term care are in Table 1.

## Longer term care: how to advance care for TBI

A key unresolved issue that impacts the care and outcome of TBI patients is how injuries are being classified. A consistent method to characterize TBI is fundamental for appropriate recruitment, application of research findings as well as to facilitate their advances for both short- and long-term care. The development and dissemination of a classification system for TBI was suggested that incorporates not only the traditional Glasgow Coma Scale (GCS) but also brain imaging as well as other prognostic biomarker and injury-related conditions with the possibility for reclassification along the further sequelae. Nine out of ten TBIs are mild (defined as a GCS of 13–15 immediately after injury) but half of all patients with mild TBI do not recover to pre-injury levels of health by 6 months [5]. Yet, compared to patients with a high-energy mechanism of injury, those with a low-energy-mechanism are about 50% less likely to receive critical care or emergency interventions [5]. Despite this, also mild TBI carries Signiant morbidity, including chronic headache and depression, which affects more than half of TBI patients [55]. Also, less than 10% of mild TBI patients receives follow-up—which would be the scientific framework to identify earlier patients at risk for incomplete recovery. Recent studies have confirmed the changing epidemiology of TBI in high-income countries where most TBIs are caused by low falls, mostly in the elderly population, often presenting with complicating co-morbidities and co-medications. However, these patients have been commonly excluded from past clinical trials and hence knowledge gaps exist regarding appropriate care for this growing patient population.

In severe TBI, the majority of acute interventions to treat have either failed or produced inconsistent results with only little improvements in survival, and if at all, only on a very low level. Rapid correction of hemostatic defects after TBI including the early administration of antifibrinolytic tranexamic acid [56] and the use of viscoelastic assays to guide hemostatic interventions [57] have been among the very few effective treatments after TBI—at least in mild to moderate TBI—and set the stage for future goal-directed and individualized therapies. Automated and dynamic analyses of blood and intracranial pressure data together with derived parameters/ratios have promoted the interest to identify TBI subgroups that may benefit from specific therapies and have introduced the concept of precision medicine into TBI which needs, of course, further refinement in the future. Blood-based protein biomarkers have potential for the evaluation of TBI including clinical decision-making and monitoring but yet one marker to date has obtained regulatory clearance to rule out the need for cranial computed tomography in mild TBI. Based upon the observations from CENTER-TBI, the second Lancet Neurology Commission on TBI published in 2022 summarized the existing knowledge gaps and provided key recommendations for subsequent research, clinical care and policy development in the context of TBI (Table 2).

**Table 2:** Priorities in research to improve trauma therapies in TBI

*Acute care*	
Develop a classification system for TBI that incorporates GCS, brain imaging and other prognostic biomarkers (egg. prognostic models across range of TBI severity, triage tools)	
Establish a research focus on mild TBI, older patients with TBI and on sex differences	
Identify subgroups that may benefit from specific therapies (precision medicine)	
*Longer term care*	
Refine, validate and implement quality indicators for TBI	
Develop an infrastructure for post-acute care (rehabilitation, treatment for chronic, long-term consequences and structured follow-up)	
identify patients at risk for incomplete recovery for modification of therapy	
*Research methodology*	
Promote the development, validation, and approval of clinical-use platforms and secure cross-platform harmonization of data and laboratory findings	
Harmonies data collection and storage on data platforms considering novel approaches to data handling, analysis and sharing	
Use of adaptive trial designs to include platform trials	
Use of Bayesian analyses	

## Discovery of novel therapies: yield and limits of trauma models

Animal models have been useful to determine dosing of medications that are used in trauma [58]. Also, animal models have advanced the understanding of the pathophysiology of trauma and the consequences of trauma on an organ level, related both to short and longer term outcomes [59]. Based on these evolving insights, animal models have been used to evaluate novel therapeutic interventions, yielding a number of potential candidate therapies intervening in a wide variety of dysregulated pathways. These include dysregulated coagulation, immune, endothelial and hormonal pathways. While not claiming to be comprehensive and acknowledging that not all research efforts can be listed here, Table 3 summarizes some interventions that have shown benefit in experimental models of polytrauma, which have not been tested yet in clinical trials.

Despite this however, none of these candidate therapies has translated into use in clinical practice as of yet. An important reason is that clinical testing lags behind, undoubtly related to high costs and resources of performing clinical trials. However, there are also therapies that have shown benefit in animal models of traumatic shock, while showing disappointing or incongruent results in clinical trials, such as artesunate [60, 61]. Reasons for this translational failure may be related to use of models that fail to adequately reflect human trauma. These include models that do not capture trauma-induced coagulopathy. An example may be an hemorrhagic model in which blood is withdrawn. While this creates shock, the lack of tissue injury will not induce release of mediators of trauma-induced coagulopathy. An ideal trauma model should have the following characteristics: low cost, trauma mechanisms and timeline similar to real-life patients, control of exogenous factors and control of potential biases [61, 62]. Thereby, the following are important requirements for poly trauma models: the potential for uncontrolled bleeding; (b) resuscitation and other therapeutic interventions occurring concurrently with hemorrhage; (c) significant soft-tissue injury to approximate the post-injury inflammatory state; and (d) the severity of trauma with lethality that closely resembles clinical situations [63, 64]. However, while models are useful to detect potential effective therapies, these therapies are unlikely to benefit all types of patients. For example, there are marked differences in the coagulation system as well as in the response to injury between males and females, as well as between the young and the elderly. Also, the existence of global genetic differences in patient responses to trauma as well as to treatments are probably also relevant. All of these facets are unlikely to be accounted for using animal models. Therefore, proof of efficacy of novel therapies remains in clinical trials, which should be as personalized as possible by accounting for these differences.

## Implementing use of biomarkers and phenotypes: improving trauma trial design

Clinical trials in severely injured trauma patients have been challenging to perform due to the inability to obtain written informed consent. This challenge has been overcome in areas where the informed written consent process can be replaced by regulatory approval process allowing a trial to be performed based on certain criteria where the benefits of the trial to the patient population may outweigh the ethical requirement of obtaining consent. Exception from informed consent has been successfully implemented in a number of recent critical trauma trials [65–68].

Optimal trauma trial design would incorporate biologically-driven inclusion criteria to enroll subjects most likely to benefit from the therapies being examined. To achieve this goal, we must first have a more complete understanding of dysregulated pathways to allow for determining candidates for interventions. We then need to develop assays that can accurately and rapidly assess for those targeted pathways within the areas of shock, endothelial, immune, and hemostatic function. What is available now for bleeding, include point of care lactate, base deficit, INR, and viscoelastic assays, which all have limitations.

An example of how biologically-driven inclusion criteria can be incorporated is the FEISTY trial (NCT05449834), which compares cryoprecipitate and fibrinogen concentrates in bleeding trauma patients where reduced fibrinogen function is part of the inclusion criteria. If biologically-driven inclusion criteria is not used and therapies are given empirically, a number of patients will not benefit since they did not need the therapy based on their pathophysiology. This increases the risk of a trial that does not show a difference in outcomes and a therapy that is potentially beneficial for some is discarded because of suboptimal trial design. The use of biologically-driven inclusion criteria will allow for the study of interventions only in specific populations that have a potential for benefit from the intervention. Another example is the use of assays related to platelet function as inclusion criteria of platelet containing products. For example, a recent study suggested that whole blood was most beneficial in subjects with a maximum clot firmness of < 60 mm [69]. A trial of whole blood that only included subjects with low platelet function would be an efficient method to determine if it was superior to other blood products. A trial could also use adaptive trial design (enrichment) to examine different thresholds of platelet function to determine the optimal laboratory-based indication. A current limiting factor is that many hemostasis assays do not yield results rapidly and delay in care when an intervention is indicated may make certain therapies less effective.

Multicenter trauma patient registries could also be helpful in determining expected frequency of specific cohorts and outcomes, but they must have monitoring that ensures high data quality to confirm accuracy and they must include all important variables, such as physiologic data related to shock, endothelial, immune and hemostasis to allow for hypothesis generating studies that will inform clinical trials. As an example, blood samples taken at regular intervals after trauma allow for measurements of hemostatic balance. Such measurements may inform trials on the optimal timing of start of thrombosis prophylaxis, or on start of prophylaxis based on biomarkers. Most trauma registries do not meet these criteria due to lack of funding required to collect a robust amount of data and to ensure data quality. This is an important limitation to overcome to allow for the optimal design and execution of clinical trials. Not only should databases be complete, they should also be standardized and accessible, to allow for the use of data science methods for analyses of all data that are derived from observational cohorts as well as trials.

Classically, randomized controlled trials use trial designs that are: 1. Determined and fixed before the trial starts and fixed during the execution of the trial, based upon data that may or may not reflect outcomes that will occur during the execution of the trial leading to inaccurate estimates of the frequency of the primary outcome 2. Typically compare two study groups with equal distribution between groups and 3. Use a frequentist statistical approach to analyze data. This standard approach limits the incorporation of new data that is generated during the trial, flexibility in altering trial design, and uses an analytic approach that is restrictive for data interpretation.

The incorporation of adaptive trial designs recently has in many ways improved study methodology if applied appropriately. A recent review of adaptive trial design [70] suggests that “An adaptive trial is designed to take advantage of the stream of information acquired during the study period to trigger specific changes or adaptations in the trial structure according to prespecified data-driven decision rules [71]. Changes that may be triggered include altering the number of treatment arms, the dosage of pharmaceutical agents, or the randomization proportions; the early termination of an arm or subpopulation for a demonstration of efficacy, futility, or harm; or the restriction of the overall study population to focus on subjects who appear to be benefiting most from the experimental therapies (termed “enrichment”). Response-adaptive randomization, in which future subjects are preferentially randomized to the arm(s) that appear most promising based on current data, can improve both the statistical performance and the ethical balance of the trial [72–75]. Trauma is an ideal setting in which to apply adaptive approaches as patient-centered outcomes are often measured in hours to days, leading to a more rapid accrual of information. Advances in Bayesian statistical methodology, including computationally intensive approaches, have facilitated adaptive trial design because the Bayesian inferential model is well suited to analyzing results when changes in the structure of the trial have occurred.” The statistical and ethical considerations have also been recently reviewed which indicate adaptive platform trials with Bayesian analyses are feasible and can be appropriate to utilize for severely injured trauma patients [76, 77].

Adaptive trial design has been successfully used in the critically ill to test COVID-19 therapies [78–83]. The MATIC-2 trauma trial (NCT06070350) makes use of an adaptive platform design to compare whole blood to component therapy, and TXA to placebo in children with traumatic bleeding. The use of platform trials with response adaptive randomization can improve the efficiency and economics of trauma trials and should be seriously considered instead of continuing to study one therapy at a time in a rigid manner that may not be translatable since severely injured trauma patients receive a bundle of care. Conventional trial design cannot adequately dissect the complexities of the bundle of acute trauma care, and this can only be evaluated with adaptive trial designs where Bayesian analyses allow for the incorporation of the clinical context to determine if the results are clinically and statistically significant [70, 76, 77]. In addition, recent success in utilizing biorepositories from RCTs in trauma to identify specific cohorts of patients who benefit from the intervention highlight the need to pair these advances in trial design with robust sampling and repository storage for assessment of mechanisms and heterogeneity of treatment effect on important clinical outcomes [15, 84, 85].

An important point in trial design is the choice of the primary outcome. Mortality is a relevant outcome, but also the most difficult one to alter with a single intervention. In order to address the ambiguity of appropriate primary outcomes, the NIH and US Department of Defense sponsored a workshop in 2019 that developed a consensus statement for recommendations of primary outcomes for clinical trials of hemostatic agents [86]. In patients with traumatic injury, excluding isolated traumatic brain injury and burn injury, the recommended primary outcome was 3–6-h all-cause mortality for hemostatic interventions, with robust evaluation of late safety-related outcomes such as 28 day mortality. In children with life-threatening traumatic hemorrhage, a primary outcome of death at either 6 or 24 h was recommended. For patients with intracranial hemorrhage the recommended primary outcome of trials of hemostatic agents was a global patient-centered clinical outcome scale measured between 30 and 180 days after the event.

**Table 3:** Candidate therapies found effective in models of severe trauma with or without bleeding, which have not been tested (yet) in clinical trials

Target	Compound/intervention	References
Oxygen carriers	Human hemoglobin	[87, 88]
	Perfluorocarbons	[89]
Platelet dysfunction	Anti-HMGB-1	[90]
	Synthetic platelet (particles)	[91, 92]
	Cold or frozen platelets	[93]
	Platelet vesicles	[94]
Endothelial permeability	rhADAMTS13	[95]
	Plasma	[95]
	Albumin	[96]
	Tyrosine kinase inhibitors	[97]
	Vasculotide	[98]
	Heparan sulfate	[99]
	Antithrombin	[100]
	Adiponectin	[101]
	(Vesicles from) stem cells	[102]
Overshoot damage molecules	Hexadimethrine bromide	[103]
	DNAse-1	[104]
Overshoot inflammation	JAK inhibitor Baricitinib	[105]
	Macrophage migration inhibitor ISO-1	[106]
	Lipid mediator Resolvin D1	[107]
	NADPH oxidase inhibitor diphenylene iodonium	[108]
	Complement activation inhibitor nomacopan	[109]
Hormones	Estrogen	[110]
Blood purification techniques	Hemoadsorption	[111]

For studies that may lack sufficient power for this outcome, a combined clinical and radiographic endpoint (such as poor outcome associated with intracranial hematoma expansion) was an acceptable second choice. From a biological perspective, it makes sense to choose an endpoint that is not too far downstream from the pathway which is modulated by the intervention. An example of a reasonable outcome of an anti-coagulant intervention is time to containment of bleeding, or transfusion need. Reasonable outcomes of an intervention targeting hyper-inflammation may be organ failure. These examples are considered interim-outcomes, which, when the effect is large enough, may translate to patient-related outcomes, which has aspects of physical, mental, social, and cognitive health, as well as quality of life. Taken together, primary outcomes can differ per intervention per trial.

## Conclusion

Improvement of outcome of trauma involves many aspects, which are related not only to the evolving trajectory of trauma which comes with different needs, but also relates to the many different aspects of care, involving multiple disciplines. From a translational outlook aiming to bring new therapies and approaches to the bedside, key research priority questions were formulated. Central to this aim is the importance of improved understanding and recognition of dysregulated pathways, which should inform biomarker-driven adaptive clinical trials (Fig. 1). In summary, the future of trauma research needs to focus on determining the right therapy for the right patient at the right time.

## Data Availability

Not applicable.

## Abbreviations

TBI: Traumatic brain injury
LMWH: Low molecular weight heparin
RBCs: Red blood cell concentrates
RCTs: Randomized controlled trials
